# Soap and water cleaning versus bleach-based cleaners for eliminating SARS-CoV-2 infection

**DOI:** 10.4102/jphia.v16i2.612

**Published:** 2025-01-29

**Authors:** Ekong E. Udoh, Ubong A. Udoh, Abiodun Egwuenu, Ekpereonne B. Esu, Aruk Eteng, Faithman E. Ovat, Uduak Okomo, Olabisi Oduwole, Joseph Okebe, Martin Meremikwu

**Affiliations:** 1 Department of Paediatrics, Faculty of Clinical Sciences, University of Uyo Teaching Hospital, Uyo, Nigeria; 2 Cochrane Nigeria, Institute of Tropical Diseases Research and Prevention, University of Calabar Teaching Hospital, Calabar, Nigeria; 3 Department of Medical Microbiology and Parasitology, Faculty of Basic Clinical Sciences, College of Medical Sciences, University of Calabar, Calabar, Nigeria; 4 Nigeria Centre for Disease Control and Prevention, Abuja, Nigeria; 5 Charité Universitätsmedizin, Berlin, Germany; 6 Department of Public Health, Faculty of Allied Medical Sciences, University of Calabar, Calabar, Nigeria; 7 Faculty of Health and Demographic Surveillance System, University of Calabar, Calabar, Nigeria; 8 Medical Research Council Unit, The Gambia at London School of Hygiene and Tropical Medicine, Fajara, Gambia; 9 Faculty of Medical Laboratory Science, Achievers University, Owo, Nigeria; 10 Department of International Public Health, Liverpool School of Tropical Medicine, Liverpool, United Kingdom; 11 Department of Pediatrics, Faculty of Clinical Sciences, University of Calabar Teaching Hospital, Calabar, Nigeria

**Keywords:** soap and water, bleach-based cleaners, SARS-COV-2 infection, households, community settings

## Abstract

**Background:**

Households and community settings are important hubs for the transmission of severe acute respiratory syndrome coronavirus 2 (SARS-CoV-2). As understanding of viral transmission improves, infection prevention and control (IPC) policies need to be updated.

**Aim:**

To compare the effectiveness of soap and water alone to bleach-based cleaners in eliminating SARS-CoV-2 infection in households and community settings.

**Setting:**

We conducted a virtual search through the Cochrane Central Register of Controlled Trials (CENTRAL), Cochrane database of systematic reviews, PubMed, EMBASE, and Effective Practice and Organisation of Care (EPOC).

**Methods:**

We assessed studies which compared the effect of soap and water cleaning on SARS-CoV-2 among humans to that of bleach-based cleaning, both in households and communities. We prioritised systematic reviews and randomised studies and only included other study designs, such as laboratory studies, which had interventions of relevant interest.

**Results:**

We retrieved 1192 articles from the search. We summarised evidence from three laboratory studies as there were no randomised controlled trials (RCTs) or comparative effectiveness studies that met our inclusion criteria. Indirect evidence suggests that soap and bleach-based cleaners were effective at different concentrations. Substantial heterogeneity between the cited studies precludes any inference on effectiveness in reducing risk of SARS-CoV-2 infection in humans. Both interventions remain important components of IPC measures.

**Conclusion:**

There was no evidence for comparison of soap and water versus bleach-based cleaners against SARS-CoV-2 in humans in household and community settings. Indirect evidence shows both interventions to be effective against the virus.

**Contributions:**

Primary studies addressing this critical question are required to guide public health recommendations and policies.

## Introduction

Severe acute respiratory syndrome coronavirus 2 (SARS-CoV-2) is a highly infectious virus responsible for the coronavirus disease 2019 (COVID-19) pandemic with nearly 650 million cases and over 6.6 million deaths reported globally in 2022.^1^ Transmission of the virus occurs directly through aerosols spread by infected persons while coughing, sneezing or speaking in close quarters and indirectly through contact with contaminated high-touch surfaces and materials such as walls, floors, windows, door handles, chairs, faucets and light switches.^2,3^ Transmission of SARS-CoV-2 in households and community settings depends on various factors, which include the prevalence of SARS-CoV-2 carrier within the community, the risk of depositing expelled viral particles on surfaces and the level of contact between people and contaminated surfaces.^4^ Thus, households and community meeting places such as public buildings, community centres, markets and transport hubs are potentially important sites for SARS-CoV-2 transmission.^5^

Fomite-mediated transmission of SARS-CoV-2 poses an important public health risk as the virus can survive on surfaces and objects for hours to days.^6^ It is estimated that 80% of common infections are spread by dirty hands, and frequent hand washing could significantly reduce the risk of acquiring colds, influenza, and other infections.^7^ Cleaning of high touch transmission surfaces in households and community will reduce the likelihood of transmission of the virus in these settings.^8^

Cleaning and decontaminating surfaces that are in contact with infected material is critical in breaking the cycle of transmission of the SARS-CoV-2 virus.^9^ The most common intervention involves cleaning with soap and water to physically remove dirt and other organic matter on contaminated surfaces.^10^ Other cleaning agents such as hypochlorite-based products are used in households and are effective against several common pathogens.^11^ The concentration and contact time, method of application, ease of use, product stability, type of surface and risk of adverse effects are important considerations for the use of cleaning products.^8^

The SARS-CoV-2 virus has a lipid envelope containing structural proteins critical for the assembly of virus-like particles.^12^ Soaps, detergents, and other lipid solvents such as sodium hypochlorite (bleach) dissolve this envelope, disrupting the virus’s ability to bind to host cells.^4,13^

Interest and demand for cleaning agents and disinfectants active against viruses have increased dramatically since the outbreak of the COVID-19 pandemic.^14^ However, cleaning products may result in health risks such as itching, redness, dryness and sores of the skin; irritation of the eyes, tearing and impaired vision. Methanol-based disinfectants are associated with headache, dizziness, ataxia, numbness while some quaternary ammonium compounds increase the risk of developing asthma, chronic obstructive pulmonary disease, infertility and impaired brain development in children.^6,7,14,15,16,17^ In view of the adverse effects associated with cleaning products, it is important to determine products that are effective but less hazardous for infection prevention and control (IPC) practices in the context of COVID-19.^8^ This review compared the evidence for using soap and water versus bleach-based cleaners for eliminating SARS-CoV-2 in households and community settings.

### Objectives

The main objective was to compare effectiveness of soap and water cleaning alone to bleach-based cleaners in eliminating SARS-CoV-2 infection in households and community settings.

The sub-objectives were to:

- determine the frequency of cleaning high-touch surfaces in community settings in the context of COVID-19
- examine the ideal cleaning products for use in households and community settings to eliminate SARS-CoV-2 infection.

## Methods

### Criteria for considering studies for this review

#### Inclusion criteria

In the order of priority, we considered systematic reviews, randomised controlled trials, cohort studies, case-control studies, controlled before-and-after studies (CBA) and interrupted time series (ITS). Where these were not available, laboratory-based experimental studies that applied the interventions of interest were considered for indirect evidence.

We considered studies that included high touch transmission surfaces in households and community settings. Where studies on households and community settings were not available, we used data from laboratory studies to generate indirect evidence on the effectiveness of the interventions.

We considered studies that focused on soap and water or detergents versus bleach-based disinfectants. We also considered studies with multiple interventions as long as they included soap and water or bleach-based products.

The primary outcome of the review was the level of SARS-CoV-2 infection among humans in household and community settings or virucidal efficacy from laboratory studies, following cleaning with soap and water alone compared to bleach-based disinfectants. Other outcomes were levels of surface decontamination following cleaning with either soap and water alone compared to bleach-based disinfectants, frequency of cleaning high-touch surfaces in community settings in the context of COVID-19 and adverse events of the cleaning products on the skin and any other organ of the body.

### Search methods for identification of studies

We searched the following databases: The Central Register of Controlled Trials (CENTRAL) and Cochrane Database of Systematic Review; PubMed, EMBASE and EPOC (The Effective Practice and Organisation of Care) for studies conducted in households and community settings between 01 January 2020, and 31 August 2022. We also searched the reference lists of retrieved full-text studies on similar topics for additional relevant studies. No language restrictions were applied.

### Search strategy

A combined search strategy was used for this review (see Appendix 1).

### Selection process

**FIGURE 1:**
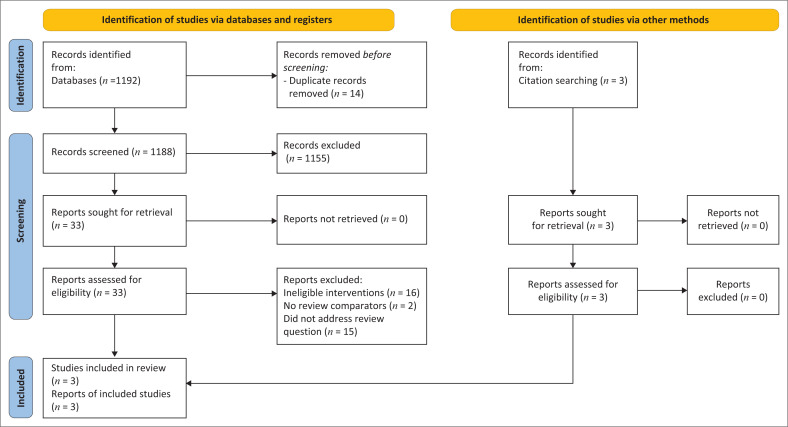
Preferred Reporting Items for Systematic Reviews and Meta-Analyses (PRISMA) flow diagram of identified studies.

Two review authors (U.A.U. and A. Egwuenu) independently screened the titles and abstracts from the literature search output for potentially relevant studies using the defined eligibility criteria. We found no study that evaluated the effect of soap and water versus bleach-based disinfectants among humans in household or community setting, or their effect on decontamination of surfaces in household or community setting. However, the reference list of two systematic reviews identified from the search had experimental studies that evaluated the effect of the intervention in laboratory settings. As a result of the absence of human studies, we included experimental studies that evaluated the effect of the interventions in laboratory setting. Differences in the classification of articles were resolved by discussion with a senior reviewer. The excluded studies and the reasons for their exclusion were noted. We used a Preferred Reporting Items for Systematic Reviews and Meta-Analyses (PRISMA) guideline and flow diagram to report the search and selection of studies^18^ (Figure 1).

### Data items

The primary outcome measure of this review was SARS-CoV-2 infection in humans, while the secondary outcomes were the frequency of cleaning of high-touch surfaces and an ideal cleaning agent for household and community in the context of SARS-CoV-2 infection. The outcome items were virucidal efficacy of the interventions, which was assessed either as log_10_ reduction of SARS-CoV-2 viral titre or as detection or non-detection of the virus on the surfaces. The log reduction was based on the 50% median tissue culture infectious dose (TCID50), which is an end point dilution assay used to measure infectious viral titre. The viral log reduction was reported along with the corresponding standard deviation. Data on the outcome measures were extracted from both the intervention and comparator arms.

### Data extraction and management

Data extraction was performed independently by two review author pairs (U.A.U. and A. Egwuenu) and (A. Eteng and F.E.O.). The extracted data included the title of the study, journal, year of publication, publication status, study setting, country, study location and study design. The review authors extracted data on the type of intervention, nature of the surfaces tainted with SAR-CoV-2, the baseline viral log load, percentage concentration of the products, and contact time (seconds/minutes) of the products on the surfaces.

### Study risk of bias assessment

Two review authors (A. Egwuenu and F.E.O.) independently assessed the risk of bias of the included studies using the Office of Health Assessment and Translation (OHAT) Risk of Bias Rating Tool.^19^ The OHAT rating tool is used to assess the risk of bias in randomised and non-randomised studies, analytical studies, descriptive studies and experimental studies. The tool has 11 domains with questions grouped to address six types of bias (selection, confounding, performance, attrition and exclusion, detection and selective reporting). The results were graded as low risk (++), probable low risk (+), probable high risk or not reported (-) and high risk (- -) (Table 2). Disagreement in judgement between the review authors was resolved by a co-author (E.E.U.).

### Measures of treatment effect and data synthesis

We did not find any study that met our inclusion criteria with respect to effect of soap and water versus bleach-based disinfection among humans in household or community setting or their effect on surface decontamination in similar settings. However, we found some laboratory-based studies that provided indirect evidence. We presented the effect of the intervention as a narrative because it was not possible to combine the data in the meta-analysis.

## Results

The search returned 1192 articles of which four were duplicates. After the titles and abstracts were screened, 33 articles were selected for full-text review. However, none of these studies met the eligibility criteria for inclusion in the review. During the screening, we identified two systematic reviews (Bedrosian et al.^20^ and Viana Martin et al.^21^) that addressed similar questions as our review although no RCT or comparative effectiveness study in the systematic reviews met the criteria for inclusion in our review. Bedrosian et al.^20^ summarised a total of 78 studies; 35 were on environmental presence of SARS-CoV-2, 16 were on surface stability of SARS-CoV-2 and 27 were on surface disinfection. Viana Martin et al.^21^ reviewed 64 studies, of which 28 focused on disinfection techniques for environmental surfaces, 16 examined disinfection methods for biological surfaces, 4 studied disinfection approaches for airborne coronavirus, and 16 explored methods for reconditioning personal protective equipment.

From the two systematic reviews, we identified three laboratory experimental studies on disinfection methods for environmental surfaces that were considered to provide indirect evidence for the effectiveness of reducing SARS-Cov-2 infection in humans from contaminated surfaces. The remaining results included a description of the findings of these studies (Figure 1).

### *Indirect* evidence on soap and water versus bleach-based products

Three laboratory-based studies were included to provide indirect evidence on the effectiveness of soap and water compared to bleach-based products in reducing the risk of SARS-CoV-2 transmission.^22,23,24^ The studies were conducted in China and the United States (US).^22,23,24^

The study by Chin et al.^23^ compared the virucidal effect of hand soap solution (1:49 dilution) versus household bleach on suspensions of 1:49 and 1:99 dilutions over 30 min. Chan et al.^22^ assessed the virucidal efficacy of liquid hand soap (biodegradable amphoteric surfactants) versus bleach (10% sodium hypochlorite) and disinfection solution (sodium hypochlorite 0.002% and 0.013% hypochlorous acid) at 5 min and 10 min post-exposure. Ijax et al.^24^ evaluated the effect of bar soap (para-chloro-m-xylenol: PCMX 0.09 g in 100 g of solution) and antiseptic solution (para-chloro-m-xylenol: PCMX ~0.12%) versus a dilutable cleaner (3.6 g of sodium chloride in 100 g of solution) and bathroom cleaner (0.4 g of sodium hypochlorite in 100 g of solution) at different contact times (Table 1).

**TABLE 1:** Description of studies used in determining indirect evidence of effectiveness.

Study ID	Setting	Aim of study	Intervention	Control	Contact time (s)	Results
Chan et al.^22^	Laboratorystudy	To investigate the efficacies of various home disinfectants against SARS-CoV-2	Liquid hand soap	Disinfecting solution: 10% bleach and dermo docyn (bleach-based disinfectant -0.002% sodium hypochlorite and 0.013% hypochlorous acid)	1 min and 5 min	Liquid hand soap achieved ≥ 2.00 ±1.56 log10 reduction in 1 and ≥ 2.25 ± 0.00 log_10_ reduction in 5 min. 10% bleach achieved ≥ 3.25 log_10_ reduction in 1 min and 5 min while dermo docyn achieved 2.30 ± 0.50 and 3.75 ± 0.43 log_10_ reduction in 1 min and 5 min, respectively.
Chin et al.^23^	*In vitro* Laboratorystudy	To test the virucidal effects of disinfectants	Hand soap solution (1:49)	Household bleach (1:49) Household bleach (1:99)	5 min, 15 min and 30 min	SARS-CoV-2 was undetected at all time points except for hand soap solution (1:49) at 5 min.
Ijax et al.^24^	*In vitro* Laboratorystudy	To evaluate the efficacies of formulated microbicidal actives against alpha- and beta-coronaviruses, including SARS-CoV-2.	Bar Soap: PCMX (0.090% w/w) Antiseptic liquid (p -chloro-m-xylenol: PCMX) Tested at an active concentration of ~0.12%	Dilutable cleaner: Sodium hypochlorite (3.6% w/w) Bathroom cleaner: Sodium hypochlorite (0.40% w/w)	5 min	Virucidal efficacies (≥ 3 log_10_ to ≥ 6 log_10_ reduction) were displayed within 30 s to 5 min.

Please see the full reference list of this article Udoh EE, Udoh UA, Egwuenu A, et al. Soap and water cleaning versus bleach-based cleaners for eliminating SARS-CoV-2 infection. J Public Health Africa. 2025;16(2), a612. https://doi.org/10.4102/jphia.v16i2.612, for more information

SARS-CoV-2, severe acute respiratory syndrome coronavirus 2; w/w, weight of solvent in weight of solution; PCMX, para-chloro-m-xylenol; ID, identification.

### Risk of bias in studies

Potential sources of bias were graded as low risk (++) if there was direct evidence in the study to indicate that the investigators applied standard procedures or practices for a given domain, it was reported as probable low risk (+) if there was indirect evidence to support the use of standard procedures or practices, probable high risk if indirect evidence suggests that best practices were not applied or what was done was not reported (-) and high risk (- -) if there was evidence in the study that suggests that standard procedures or practices were not applied for the domain.^19^

There was information in the included studies to decide on the Risk of Bias (ROB) for most of the domains assessed except for the domain on ‘Were research personnel blinded to the study group during the study?’. None of the authors provided any information on this. The ROB of the included study was low for most of the evaluable domains (Table 2).

**TABLE 2:** The office of health assessment and translation risk of bias rating tool for human and animal studies.

Description of included studies	Chan et al.^22^	Chin et al.^23^	Ijaz et al.^24^
Study design	*In vitro* study	*In vitro* study	*In vitro* study
	Experimental study	Experimental study	Experimental study
Was administered dose or exposure level adequately randomised?	Not Applicable	Not Applicable	Not Applicable
Was allocation to study groups adequately concealed?	Not Applicable	Not Applicable	Not Applicable
Were experimental conditions identical across study groups	++ Low risk	++ Low risk	++ Low risk
Were research personnel blinded to the study group during the study?	Not Reported	Not Reported	Not Reported
Were outcome data complete without attrition or exclusion from analysis?	++	++	++
Can we be confident in the exposure characterisation?	++	++	++
Can we be confident in the outcome assessment (including blinding of assessors)?	+	+	+
Were all measured outcomes reported?	++	++	++
Were there no other potential threats to internal validity	++	++	++

Note: Potential source of bias was graded as low risk (++), probable low risk (+), probable high risk or not reported (-) and high risk (- -). Please see the full reference list of this article Udoh EE, Udoh UA, Egwuenu A, et al. Soap and water cleaning versus bleach-based cleaners for eliminating SARS-CoV-2 infection. J Public Health Africa. 2025;16(2), a612. https://doi.org/10.4102/jphia.v16i2.612, for more information

### Certainty of evidence

The certainty of evidence of the virucidal effect of the interventions on SARS-CoV-2 contaminated surface was not assessed using the Grading of Recommendations, Assessment, Development and Evaluation (GRADE) approach. However, it was considered to be very low because it was based on laboratory studies, which are generally of low methodological quality. Thus, this review provides an indirect evidence on the effect of the interventions in households and community settings.

### Results of individual studies

#### Effect of soap and water cleaning alone versus bleach-based cleaners

The assessment of the viral inactivation was determined by the log_10_ reduction of SARS-CoV-2 or its recovery from the surface at a predetermined time after the application of the microbiocidal agent. Viral recovery from surfaces assessed over 30 min of application of hand soap (1:49) versus household bleach (1: 49 and 1: 99) was undetectable, except at the 5 min assessment in which viral recovery of 3.6 log_10_ TCID50/mL was reported in surfaces cleaned with hand soap (1:49) solution Chin et al.^23^

Chan et al.^22^ reported 2.00 ± 1.56 and 2.25 ± 0.00 log_10_ SARS-CoV-2 reduction in 1 min and 5 min, respectively, for liquid hand soap, ≥ 3.25 log_10_ reduction in 1 min and 10 min for bleach (10% sodium hypochlorite) and 2.30 ± 0.50 and 3.75 ± 0.43 log_10_ reduction in 1 min and 5 min, respectively, for dermo docyn (0.002% sodium hypochlorite and 0.013% hypochlorous acid).

Ijax et al.^24^ reported a log reduction of ≥ 3 to ≥ 6 log_10_ within 30 s to 5 min using different soaps (bar soap and antiseptic liquid) and bleach-based disinfectants (3.6% w/w sodium hypochlorite and 0.40% w/w [weight of solvent in weight of solution] sodium hypochlorite) in suspension. The different products achieved ≥ 4.1 log_10_ reduction in the SAR-CoV-2 titre at 0.5 min to 5 min contact time.

#### Frequency of cleaning high-touch surfaces in the context of COVID-19

These were laboratory-based studies. They did not have information that could infer the frequency of cleaning high-touch surfaces in household or community settings.

#### The ideal cleaning products for use in households and community settings

The included studies did not contain the information needed to determine the ideal agent for household and community use in the context of SARS-CoV-2.

## Discussion

The core IPC measures against the spread of the SARS-CoV-2 virus are social distancing, hand washing, wearing of face masks and disinfection of surfaces and materials.^25^ There are many types of disinfecting agents used worldwide for cleaning and disinfection, in healthcare settings. However, the spread of the coronavirus had informed the extended usage of these agents in households and community settings.^26,27^ As the understanding of the SARS-CoV-2 virus transmission improves, it is important to review and update public health recommendations on IPC measures aimed at reducing the spread of the virus.

We did not find any study that addressed the effect of these interventions on the risk of SARS-CoV-2 infection in household and community settings. We identified three primary studies from which we generated indirect evidence on the effect of the soap and bleach-based disinfectants in reducing SARS-CoV-2 infections in humans. These were *in vitro* laboratory experimental studies that evaluated the virucidal effect of the agents against the SARS-CoV-2 infection.

Based on the 2018 US Environmental Protection Agency (EPA) guideline, disinfectant are deemed effective if they meet the following criteria^28^: ≥ 4.8 log_10_ of infectivity per carrier be recovered from the dried virus control film; ≥ 3 log_10_ reduction in titre is observed in the presence or absence of cytotoxicity; if cytotoxicity is present, ≥ 3 log_10_ reduction in titre is observed beyond the cytotoxic level; and cell controls (cells not spiked with virus) be negative for evidence of infectivity (i.e., viral cytopathic effect or plaques). The viral inoculum in the studies included for the indirect evidence ranged from 6.50 ± 0.61 to 9.8 log_10_ plaque-forming units [pfu] mL^−1^), which was above the ≥ 4.8 log_10_ of infectivity per carrier in dried virus control film needed to evaluate the virucidal effectiveness of disinfectants based on the US EPA of 2018.^28^

Indirect evidence from three *in vitro* studies showed that commercially available soap and/or detergent demonstrated virus-killing properties with log reduction ≥ 3.25 ± 0.00 between 0.5 min and 10 min, which is above the US EPA guideline for effective disinfectant agent. Bleach-based disinfectant (dilutable cleaner: 3.6% sodium hypochlorite) and bathroom cleaner (0.40% sodium hypochoride) achieved a viral reduction ≥ 5.10 ± 0.00 log_10_ pfu mL^−1^ by 0.5 min and 5 min, respectively, on suspension (Ijax et al.^24^). There was a reduction of ≥ 3.25 ± 0.00 log_10_ pfu mL^−1^ with the use of bleach (10% sodium hypochlorite) at 1 min which persisted up to 5 min contact time (Chan et al.^22^). This also indicates that both products are effective against SAR-CoV-2 infection.

### Limitation of evidence

The evidence on the effectiveness of the interventions in this review was based on laboratory studies as against humans residing in households or community. The behaviour of the SARS-CoV-2 virus on surfaces in controlled laboratory settings is likely to be different from the typical environmental surfaces in households and community settings.

Evidence on frequency of cleaning or disinfection of high touch environmental surfaces on the transmission of SARS-CoV-2 infection in households and community settings could not be generated because of lack of necessary data in the included studies. However, some factors that might influence the frequency of cleaning or disinfection of high touch surfaces include nature of the surfaces, degree of soilage, frequency of contamination, efficacy of the virucidal agent and environmental conditions such as temperature and relative humidity. Likewise, evidence on the ideal cleaning product for households and community settings in the context of SARS-CoV-2 infection could not be generated because of inadequate information.

### Implication for practice

Indirect evidence from *in vitro* laboratory studies indicates that soap and/or detergent and bleach-based disinfectants have good virucidal property with ≥ 3.25 ± 0.00 log_10_ reduction of SARS-CoV-2 titre between 0.5 and 10 min surface contact time. The virus-killing properties of both products meet the US EPA guideline for effective disinfectant agent. These products can be used to clean or disinfect contaminated household and community surfaces in the context of SARS-CoV-2.

### Implication for future research

There is presently no direct evidence on the effectiveness of soap and/or detergent or bleach-based disinfectants in mitigating the spread of SARS-CoV-2 infection in households and community setting. There is need for more virucidal efficacy data for soap and/or detergent and bleach-based disinfectants against SARS-CoV-2 in human participants at household and community settings.

Well-designed robust studies on soap and/or detergents and bleach-based disinfectants that will generate reliable evidence to address public health questions on the efficacy and frequency of cleaning of high touch environmental surfaces are needed. There is also a need for data on protocols for cleaning products, stability in different environmental conditions, acceptability, usability, cost-effectiveness and adverse effect of the products to determine the ideal cleaning agent or disinfectant for use in households and community settings to mitigate the spread of SARS-CoV-2 infection.

## Conclusion

There was no direct evidence on the effectiveness of soap and water versus bleach-based cleaners against SARS-CoV-2 in household and community settings. Indirect evidence shows both interventions to be effective in eliminating SAR-CoV-2 infections. The studies were associated with some heterogeneity, which poses an important challenge on the generalisability and applicability of the evidence. Primary studies addressing this critical question are required to guide public health recommendations and policies.

## Appendix

**TABLE 1-A1:** Search strategy of study.

1.	Housing/
2.	Workplace/
3.	schools/ or schools, nursery/ or universities/
4.	‘non-medical public and private facilities’/or exp child day care centers/or libraries/or restaurants/or exp ‘sports and recreational facilities’/or transportation facilities/or museums/or public facilities/
5.	household articles/ or household products/
6.	(community or communities or home* or house* or housing or residence* or residential or workplace* or work place* or school or schools or college* or universit* or library or libraries or museum* or restaurant* or shop or shops or retail or sport* or athlet* or gym* or recreation* or transportation).ti,ab,kf.
7.	(nonmedical or nonhealth* or non medical or non health*).ti,ab,kf.
8.	or/1–7
9.	exp Health facilities/ or exp hospitals/ or exp Nursing Homes/ or Homes for the Aged/ or assisted living facilities/
10.	(hospital* or dental or clinic or clinics or nursing or ‘Old Age Homes’ or ‘Old Age Home’ or ((medical or health or healthcare) adj1 (facility or facilities or centre* or center* or office* or setting*))).ti,kf.
11.	((longterm care or long term care) adj3 (home* or facility or facilities or residen* or hous* or setting*)).ti,kf.
12.	9 or 10 or 11
13.	8 not 12
14.	Disinfection/ or exp Disinfectants/ or exp Detergents/ or Fumigation/ or Decontamination/
15.	exp chlorine compounds/ or hypochlorous acid/ or sodium hypochlorite/
16.	Quaternary Ammonium Compounds/ or Bleaching Agents/ or Hydrogen Peroxide/
17.	exp ethanol/ or exp ethanolamines/ or 2-Propanol/
18.	methylene blue/
19.	((sanitation or sanitary or housekeep* or janitor*) adj3 (worker* or staff or employee* or personnel or occupation* or team* or department*)).ti,ab,kf.
20.	(brush or brushing or clean* or disinfect* or decontaminat* or fogging or fogger* or fumigat* or mist or misting or sanitis* or sanitiz* or scrub or scrubbing or spray* or wipe or wiping).ti,ab,kf. not scrub typhus.mp.
21.	(detergent* or soap* or methylene blue or ethanol or bleach* or chlorin* or hypochlorit* or hydrogen peroxide or glutaraldehyde or formaldehyde or aldehyde or dihydrogen dioxide or hydrogen dioxide or hydroperoxide or quaternary ammonium or quaternary bisammonium or quaternized amine or hypochlorous acid).ti,ab,kf.
22.	(antiseptic* or iodine or ethanolamin* or isopropanol or isopropyl or propanol or 2propanol).ti,ab,kf.
23.	or/14–22

### Other information

#### Registration and protocol

The review protocol was published on PROSPERO (CRD42022356799).
